# Tensile and Interfacial Mechanical Properties for Single Aramid III Fibers under Dynamic Loading

**DOI:** 10.3390/polym16060804

**Published:** 2024-03-13

**Authors:** Fu Liu, Fangfang Li, Xuelei Li, Haobin Tian, Xudong Lei

**Affiliations:** 1 School of Intelligent Manufacturing and Control Engineering, Shanghai Polytechnic University, Shanghai 201209, China; liufu@sspu.edu.cn (F.L.); lifangfang@sspu.edu.cn (F.L.); lixuelei@sspu.edu.cn (X.L.); tianhaobin@sspu.edu.cn (H.T.); 2 Institute of Mechanics, Chinese Academy of Sciences, Beijing 100190, China

**Keywords:** Aramid III, single fiber, surface modification, dynamic mechanical property, IFSS, mini SHTB, failure morphology

## Abstract

In this study, the traditional mini split Hopkinson tension bar (SHTB) was enhanced for the dynamic mechanical performance testing of single fiber/resin interface of composites. Single Aramid III fibers were modified using a polyamine modification treatment. Quasi-static and dynamic tensile tests of modified single Aramid III fibers were conducted using an electronic tensile testing machine and mini SHTB. The test results indicated that the surface modification employing the Catechol-Tetraethylenepentamine (Cat-TEPA) approach had a negligible effect on the tensile mechanical properties of single Aramid III fibers. The microdroplet method was introduced to measure the dynamic interfacial shear strength (IFSS) of Aramid III fiber/waterborne polyurethane resin using a mini SHTB. The dynamic shear test results revealed an increase in the dynamic shear strength of the modified Aramid III fiber/resin interface from 36.16 MPa to 41.51 MPa. Furthermore, the Scanning Electron Microscope (SEM) photography of the modified single Aramid III fiber after debonding exhibited regular grid structures on the debonding area, which can prevent debonding between the single fiber and the microdroplet, thereby enhancing interfacial shear performance.

## 1. Introduction

The Aramid Fiber Reinforced Composite (AFRC) has been widely used in aerospace and defense industries owing to its high specific strength, high specific stiffness, and excellent anti-impact performance [1,2,3,4]. Particularly in protective structural applications, the AFRC has proven crucial to the design of body armor and bulletproof helmets due to its excellent toughness and high modulus. When subjected to impact loads, the three primary failure models of composites are fiber fracture, matrix crack, and delamination, which play pivotal roles in determining the energy absorption performance of protective structures [5,6].

Several experimental studies have recently indicated that aramid fiber is strain-rate sensitive under high-speed dynamic loads. Yao et al. conducted high-rate and quasi-static tests on aramid fabric specimens, observing increases in their tensile strength, toughness, and maximum strain with increasing strain rate [7]. Tan et al. utilized quasi-static and dynamic experimental techniques for tensile testing of Twaron^®^ fiber bundles, revealing a strain rate hardening effect on the modulus, failure strain, and stress of the fiber [8]. In addition to investigating the dynamic properties of AFRC test pieces or fiber bundles, the study of the dynamic mechanical properties of single aramid fibers is crucial. Lim et al. studied the dynamic mechanical behavior of single aramid A265 fibers using a split Hopkinson tension bar (SHTB), and found that the ultimate strength increased by 16.6% when the strain rate was increased from 0.001 s^−1^ to 1000 s^−1^ [9]. Cheng et al. investigated the dynamic ultimate strength and failure strain of single Kevlar KM2 fibers within a strain rate range of 1400 s^−1^ to 2500 s^−1^. The measured dynamic ultimate strength and failure strain were 4.04 GPa and 4.68%, respectively, both larger than the quasi-static results [10]. Lei et al. conducted quasi-static and dynamic mechanical properties tests of single Aramid III fibers using a small-scale tensile testing machine and mini-SHTB, respectively. The experimental results showed that single Aramid III fibers exhibited an obvious strain rate strengthening effect [11].

Not only does the strain rate strengthening effect of aramid fiber contribute to improved impact resistance, but also the characteristics of the aramid fiber/resin interfacial bond play a crucial role in enhancing the performance of protective structures. The debonding failure between the fiber and the resin can absorb a significant amount of energy under impact loads, highlighting the importance of the interfacial bond strength in energy absorption. Due to the high degree of crystallinity, smoothness, chemical inertness, and the poor fiber/resin interfacial bond characteristics of the surface of aramid fiber, the surface modification of aramid fiber is necessary to enhance its interfacial bond properties. In recent years, numerous techniques have emerged to improve fiber/resin interfacial bond properties, including surface coating, physical, chemical, and biological treatments [12]. LaBarre et al. modified Kevlar K129 yarns and fabrics through sonication in a solution of N-methylpyrrolidone (NMP) and multi-walled carbon nanotubes (MWNTs). Material properties tests of MWNT-treated yarns revealed an increased yarn modulus, static and kinetic friction coefficients, and pull-out forces. Low-velocity impact tests on a single ply of MWNT-treated fabrics with a negligible mass addition showed an approximately 50% increase in the ballistic limit [13]. Compared to other fiber modification methods, the dopamine surface modification method, based on a surface coating technique, offers simplicity, rapidity and environmental friendliness. It can improve the surface performance of the material, such as increasing the hydrophilicity, improving the cell attachment properties, enhancing the biocompatibility, and reducing the adhesion of cells, etc., which is widely used in the fields of biomedicine, nano-materials, and optoelectronic materials. The self-polymerization of dopamine deposits polydopamine (PDA) films on fiber surfaces, while pyrrole oxidatively polymerizes on fiber surfaces to form polypyrrole (PPy), with these coatings finding widespread applications. The PDA coating treatment stands out for its simplicity, versatility and cost-effectiveness [14,15,16]. Sa et al. proposed a combination of bio-inspired PDA deposition and epoxy grafting to prepare ultra-high molecular weight polyethylene (UHMWPE) fibers, leading to improved interfacial adhesion properties. The pull-out force increased by 67.5% [17]. However, the dopamine surface modification technique has some disadvantages and limitations. Firstly, its effectiveness depends on various factors such as the reaction conditions, dopamine concentration, and reaction time, necessitating multiple tests to determine the optimal reaction conditions. Secondly, the technique can only be conducted under alkaline conditions, which may damage or deactivate sensitive materials. Additionally, the surface modified by dopamine surface modification is thin, with a thickness ranging from a few nanometers to tens of nanometers. Thirdly, dopamine is relatively expensive, limiting its industrial application. Consequently, researchers have explored alternative techniques for surface modification. It has been found that the key to the oxidative polymerization of dopamine lies in the presence of both catechol and amino groups [16]. Wang et al. replaced dopamine with low-cost catechol and polyamine, simplifying the polymerization process by avoiding the cyclization within the dopamine molecule [18]. Zhao et al. conducted the surface modification of UHMWPE fibers using Cat-TEPA, resulting in a 44% increase in the modified fiber/resin interface shear strength [19].

In order to study the fiber/resin interfacial bonding force after surface modification and validate the effectiveness of the modification process, various test methods have been introduced to measure interfacial shear strength (IFSS). Recently, common methods include single fiber pullout [20,21], single fiber fragmentation [22,23], single fiber push-out [24,25], and the microdroplet technique [25,26]. The single fiber pullout method involves embedding a single fiber into the resin matrix, applying tension force to the upper end of the fiber, and extracting the fiber as a whole. The IFSS is ultimately determined based on the tension force, the embedding depth, and the fiber diameter. In the single fiber fragmentation method, a single fiber is first embedded into the resin matrix and then made into a dog bone-shaped specimen for tensile testing. When the matrix is subjected to tension, the force is transmitted through the interface to the single fiber. As the load increases, the fiber will exhibit multiple breakpoints, with the number of breakpoints increasing with the load. When the load increases to the point where the fiber is sufficiently short and cannot break further, reaching a state of fracture saturation, the IFSS between the fiber and resin can be obtained. A greater number of breakpoints indicates more timely stress transmission and better interface adhesion. The single fiber push-out method is generally used to test the interface properties of resin-based and ceramic-based composite materials, developed based on the single fiber pullout method. In the experiment, the interface of the composite material sample is perpendicular to the fiber axis, and an extremely sharp diamond and other punches are used to apply axially increasing pressure to the upper surface of the single fiber until the fiber is peeled off from the composite material. During this process, the extrusion load–displacement curve is recorded to assess the IFSS. However, most of these studies were conducted under quasi-static rate conditions. Due to the challenges associated with dynamic test methods, there are limited reports in the literature on fiber/resin interfacial performance tests under dynamic loading. Chu et al. utilized a modified tension Kolsky bar with a high-speed synchrotron X-ray phase-contrast imaging setup to investigate the dynamic crack initiation and propagation at the fiber–matrix interface using pull-out configuration, and found that the debonding mechanism, peak force, and interfacial shear stress remained rate-insensitive as the pull-out velocity increased from 2.5 to 5.0 m/s [27]. Li et al. employed a modified split Hopkinson pressure bar (SHPB) system to conduct push-out experiments for single fibers, noting that the maximum push-out force increased with the loading rate [28]. Tamrakar et al. developed a novel method to measure the high-rate interface properties of composites by loading a microdroplet test specimen into a modified SHTB [29]. In experiments based on the microdroplet technique, a microdroplet of the matrix resin (μm scale) is deposited on a single fiber, and then the cured microdroplet is stripped with a cutter device to obtain the IFSS by measuring the force. This technique offers advantages such as its easy sample preparation, high testing efficiency, and relatively small data dispersion [30,31].

To greatly enhance the impact resistance of AFRC-protective structures, the modification of aramid fiber is essential. Additionally, studying the tensile and interfacial mechanical properties of single aramid fibers under dynamic loading is crucial. In this study, the surfaces of Aramid III fibers were modified using the Cat-TEPA co-deposition approach, and the prepared samples were designated as Aramid III @ Cat-TEPA. The dynamic tensile strength of the single fibers and the shear strength of the fiber/resin interface were determined using an improved mini SHTB. The microstructure and debonding failure of the samples were analyzed through SEM photography.

## 2. Experiment

### 2.1. Materials and Polyamine Modification of Aramid III Fibers

Aramid III fiber bundles were provided by China Bluestar Chengrand Co., Ltd. (Chengdu, China), while waterborne polyurethane resins were purchased from ShenZhen JiTian Chemical Co., Ltd. (Shenzhen, China).

The surfaces of the Aramid III fibers were modified by Cat and TEPA in a buffer fluid of trimethylolpropane and hydrochloric acid (Tris-HCl), resulting in the formation of a cross-linked network structure. In the Cat-TEPA co-deposition reaction, both the primary amino group and secondary amino group of TEPA can participate in the cross-linked reaction, with the primary amino group being more active. When the molar ratio of Cat and TEPA is 1:1, the cross-linked product will still contain a certain percentage of the primary amino group. Increasing the proportion of TEPA can enhance the content of the primary amino group in the cross-linked product, thereby improving the interfacial strength of the fiber/resin interface and the mechanical properties of the AFRC.

**Figure 1:**
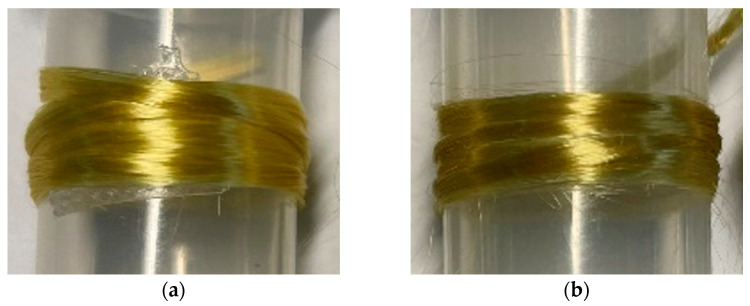
Appearance of Aramid III fiber bundles: (**a**) Before modification; (**b**) After modification.

**Figure 2:**
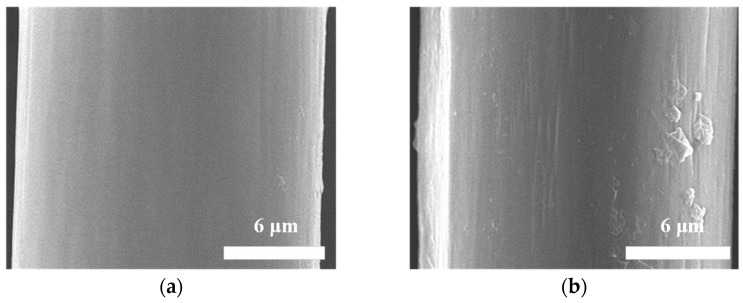
SEM morphology of a single Aramid III fiber’s surface: (**a**) Before modification; (**b**) After modification.

In this work, the molar ratio of Cat and TEPA was set as 1:4. The cross-linked reaction took 24 h, during which the color of the Aramid III @ Cat-TEPA fibers changed from yellow to dark brown with a relatively uniform distribution, as illustrated in Figure 1. Additionally, the microscopic morphology of the single fiber surface was examined by a SEM, as shown in Figure 2. Figure 2a depicts the relatively smooth surface before modification, while Figure 2b reveals a layer of the uniform coating on the fiber surface, while a few granules were observed after modification, likely resulting from attachments during the Cat-TEPA co-deposition process in the reaction fluid.

### 2.2. Tensile Test of Single Aramid III Fibers

**Figure 3:**
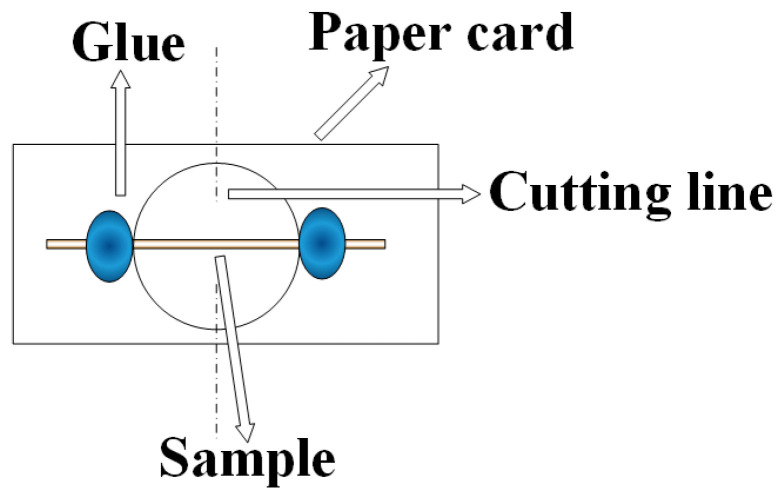
Schematic diagram of the tensile sample for a single fiber.

The preparing process and test method for single Aramid III fiber samples were based on the work of Lei et al. [11]. The effective number of samples for all experiments is 5. The single fiber samples were positioned as illustrated in Figure 3. To strictly control the gauge section of the sample, the position of the adhesive should be tightly placed against the edge of the circular hole. The samples were affixed to paper cards using ethyl cyanoacrylate glue, then were stood for more than 24 h to ensure the secure adhesion of the single fibers to the paper cards.

**Figure 4:**
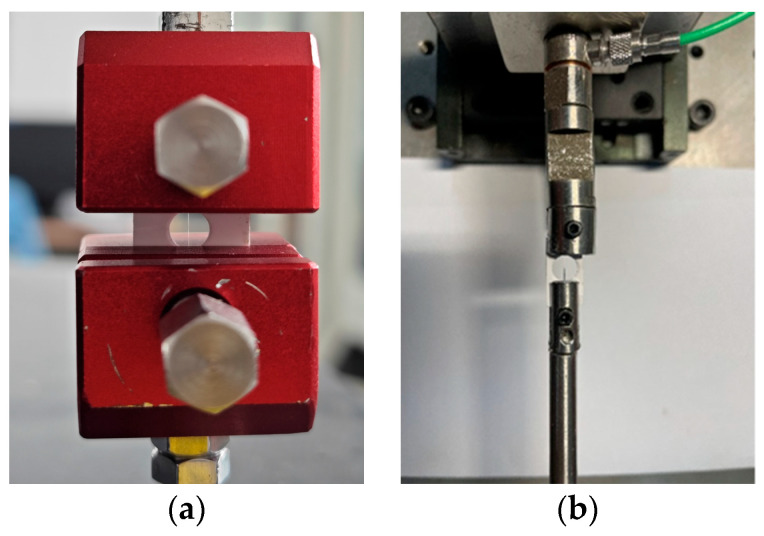
Tensile clamping: (**a**) Quasi-static tensile clamping; (**b**) Dynamic tensile clamping.

The prepared single Aramid III fiber samples were secured onto the fixtures of the test instruments, as shown in Figure 4. Figure 4a shows the quasi-static tensile clamping in the electronic tensile testing machine, while Figure 4b depicts the dynamic tensile clamping in the improved mini SHTB.

### 2.3. Shear Strength Test of Aramid III Fiber/Resin Interface

**Figure 5:**
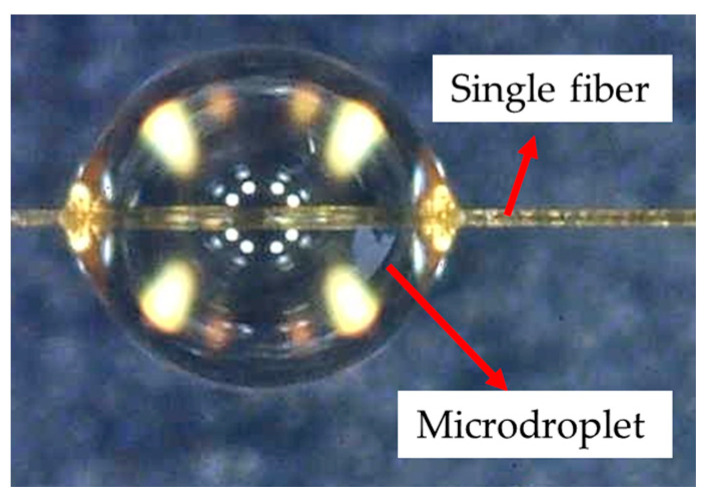
The microdroplet sample of a single Aramid III fiber.

Drawing from the studies of Miller et al. [31] and Gaur et al. [32], a dynamic microdroplet method was employed to measure the IFSS between a single Aramid III fiber and the resin. Microdroplets made of waterborne polyurethane resin were utilized, cured at room temperature for 24 h. Figure 5 shows the formed experimental samples using this method. It can be observed that the fiber was completely enwrapped by the resin, forming an elliptical resin microdroplet.

**Figure 6:**
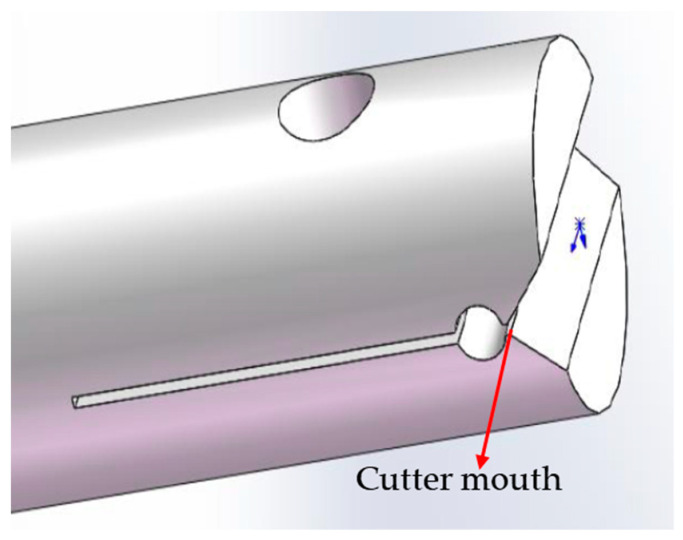
The fixture for the dynamic fiber/resin interface properties test.

**Figure 7:**
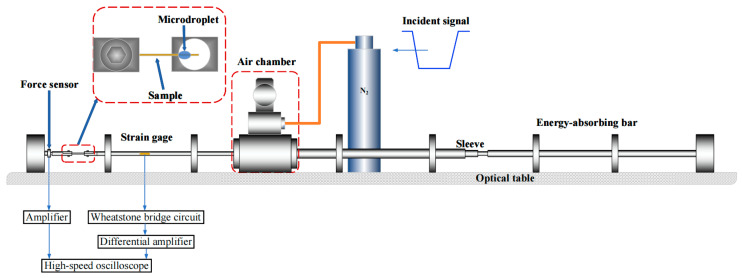
Schematic diagram of the mini SHTB device for the dynamic fiber/resin interface properties test.

**Figure 8:**
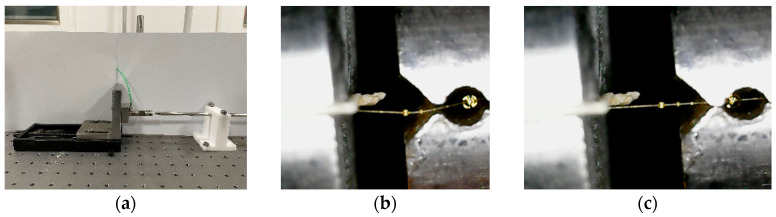
Dynamic microdroplet test: (**a**) Dynamic microdroplet testing device; (**b**) Initial clamping state; (**c**) Final clamping state.

When conducting the dynamic Aramid III fiber/resin interface test, it is important to note that the force signal resulting from interface failure is typically very weak. Therefore, a high-sensitivity piezoelectric force transducer was used instead of a transmitted bar to measure the force. Given that the diameter of a single Aramid III fiber is approximately 17 μm, it is crucial to ensure that the dimension of the cutter mouth is slightly larger than the fiber diameter to obtain accurate results. To facilitate these measurements, a dynamic test fixture was designed, as shown in Figure 6, which can achieve the goal of having the dimension of the cutter mouth be slightly larger than the fiber diameter through narrow gaps and screws. The mini SHTB utilized for dynamic fiber/resin interface properties testing is illustrated in Figure 7, which mainly consists of a high-pressure gas emission system with a pressure range of 0–1 MPa, an incident bar, an energy absorption device, a piezoelectric force sensor, a strain-measuring instrument, and an oscilloscope. In the experiment, a high-pressure gas emission system is utilized, consisting of a high-pressure gas chamber and a sleeve-type bullet. By opening the valve of the gas chamber, compressed N_2_ gas is released to propel the sleeve-type bullet to accelerate within the gun barrel. The bullet impacts a mass block fixed at the end of the bar at a certain velocity, generating a tensile wave. This wave propagates along the incident bar to one end of the clamped sample, where it loads the microdroplet specimen until interfacial shear failure occurs. It is crucial to ensure that the axis of the sample is aligned with that of the bar during fixation. During the experiment, it was found that friction between the sleeve-type bullet and the incident bar led to the premature failure of the microdroplet sample interface. Therefore, we have improved the SHTB device by adding an energy-absorbing bar and fixing its end. This method has three advantages: (1) The inclusion of the energy-absorbing bar prevents the superimposition of a primary tensile wave generated by the impact of the sleeve-type bullet with a secondary compressive wave reflected from the end of the mass block, ensuring that only the curve of the stress wave generated by impacting the energy-absorbing bar is observed. (2) It addresses the problem of the friction-induced movement of the bar, which leads to the premature interface failure of the microdroplet sample, preventing signal capture. (3) The fixed end of the energy-absorbing bar prevents the reverse movement of the incident bar after impact, thereby avoiding further impacts on the microdroplet sample and highly sensitive force sensors (if smaller range sensors are used, the impact force generated by reverse movement may damage the equipment), thus protecting the failed samples for the subsequent observation of failure morphology. To capture the rapid response of the fiber/resin interface, a piezoelectric force sensor of model Kistler 9001 (Winterthur, Switzerland) was used in the experiment, with a frequency response up to 180 kHz, and a resolution of 5 mN. The output charge signal from the piezoelectric force sensor needs to be amplified using a charge amplifier, and then the amplified signal is collected through data acquisition equipment. The model of the charge amplifier is Kistler 5015, which is a dual-mode charge amplifier. It is equipped with a high-pass filter internally and is connected to the piezoelectric force sensor via cables. The voltage corresponding to the force value can be adjusted to meet different experimental requirements. In this experiment, a 1 V voltage corresponds to a force of 0.1 N, enabling the accurate measurement of the force state of the fiber/resin interface. The output signals of the force sensor and strain measuring instrument are simultaneously collected using an oscilloscope. In the mini SHTB device, the materials of the sleeve-type bullet, incident bar, and energy-absorbing bar are 45# steel. The incident bar has a diameter of 6 mm and a length of 1500 mm; the energy-absorbing bar has a diameter of 10 mm and a length of 1000 mm; the inner and outer diameters of the sleeve-type bullet are 6 mm and 10 mm, respectively, with lengths of 200 mm, 300 mm, and 400 mm. The maximum velocity of the sleeve-type bullet can reach 10 m/s, and different tensile wave pulse widths and amplitudes are achieved by adjusting the length and velocity of the bullet. Figure 8a displays the dynamic microdroplet test device. During the experiment, the microdroplet sample was first placed into the fixture, as shown in Figure 8b. Subsequently, the force transducer was slowly moved using the knob. The cutter mouth was closed when the force transducer reached a suitable position, and the microdroplet was securely held for testing, as depicted in Figure 8c.

The IFSS *τ* of the Aramid III fiber/resin interface can be calculated as [29]:

(1) $$\tau =\frac{F}{\pi d_{f}l_{e}}$$

where *F* is the interfacial force signal of the fiber/resin interface recorded by the force sensor, *d_f_* is the diameter of the single Aramid III fiber, and *l_e_* is the length of the microdroplet. To ensure the accurate measurement of the IFSS, the size of the microdroplet must be strictly controlled so that the debonding force of the fiber/resin interface is less than the breaking force of the single fiber. Therefore, *l_e_* should satisfy the following equation [29]:

(2) $$l_{e}<\frac{\sigma d_{f}}{4\tau }$$

where *σ* is the tensile strength of the single Aramid III fiber.

**Figure 9:**
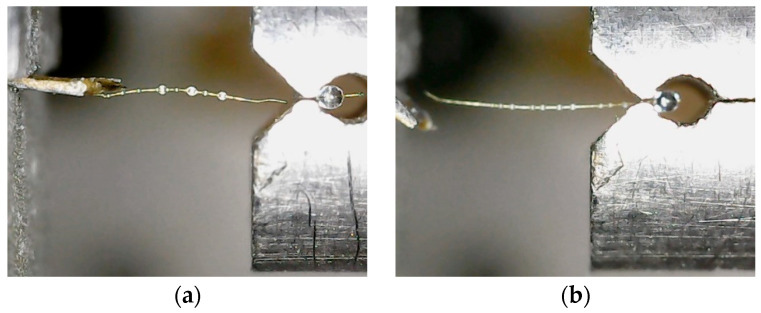
Typical invalid experimental results: (**a**) Rupture of the single fiber due to a too large microdroplet; (**b**) Fiber slid from end.

Figure 9 illustrates two typical invalid experimental results. Figure 9a presents that the single fiber ruptured because the microdroplet was too large. In this scenario, the contact length between the microdroplet and the single fiber was too long, causing the load required for interfacial shear failure between the fiber and the resin to exceed that required for fiber fracture, resulting in single fiber fracture without interfacial failure. Figure 9b depicts a situation where the single fiber slid from the end due to the poor bonding strength of the fiber/substrate interface. In this case, the bonding length between the single fiber end and the paper card was too short, leading to the bonding strength between the fiber and the resin exceeding that between the fiber end and the paper card during stretching.

**Figure 10:**
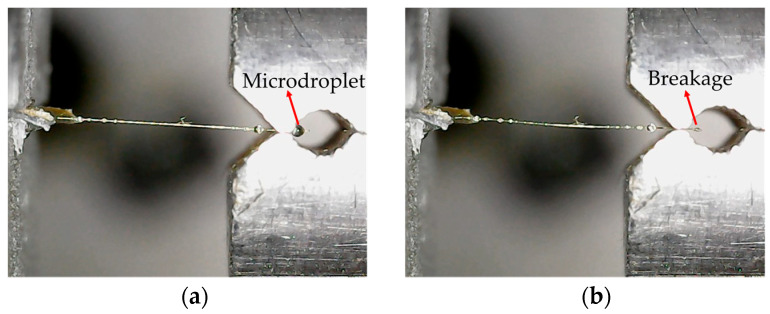
Poor curing performance of the microdroplet, leading to breakage after the experiment: (**a**) Before experiment; (**b**) After experiment.

As shown in Figure 10, it is evident that the microdroplet broke due to inadequate resin curing or the excessively large dimension of the cutter mouth. Figure 10a and Figure 10b, respectively, depict the morphology of a microdroplet sample before and after testing. It can be observed that the insufficient curing of the microdroplet resulted in its rupture after testing, rather than sliding along the fiber, and in such instance, successful measurement of the IFSS becomes unattainable.

**Figure 11:**
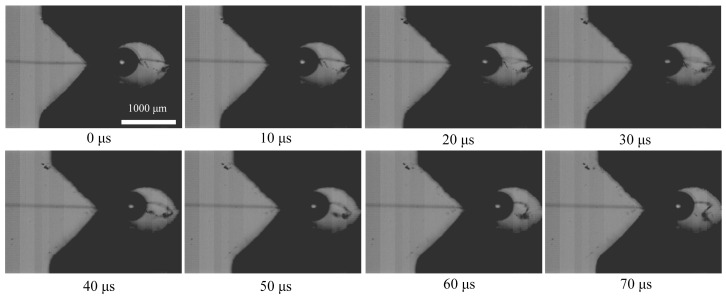
Photography of the dynamic debonding process of the microdroplet.

**Figure 12:**
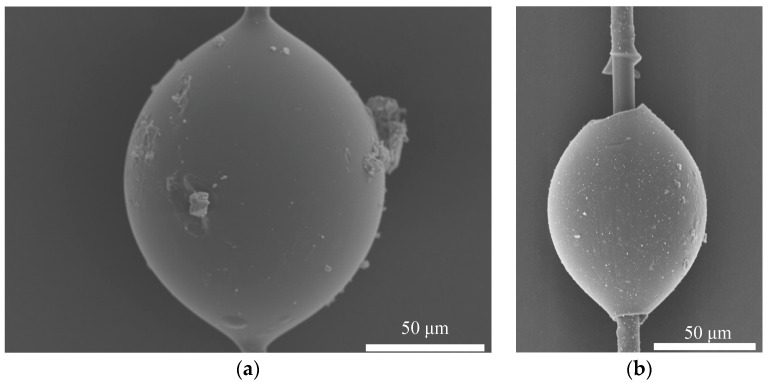
The morphology of the microdroplet sample: (**a**) Original microdroplet morphology; (**b**) Microdroplet morphology after debonding.

Figure 11 illustrates the complete process of the dynamic debonding of the microdroplet captured by high-speed photography, and the time interval between each frame is 10 μs. It can be observed that the blade contacted the resin microdroplet and peeled it off along the single fiber. The intact single Aramid III fiber observed during the tensile process confirms the validity of the test method. Figure 12 displays the morphology of the microdroplet sample before and after the test. Initially, as shown in Figure 12a, the bonding between the microdroplet and single fiber was intact. However, as the shear stress of the fiber/resin interface exceeded the shear strength, the microdroplet was completely stripped due to debonding, as depicted in Figure 12b.

## 3. Results and Discussion

### 3.1. Tensile Strength of Single Aramid III @ Cat-TEPA Fibers

**Figure 13:**
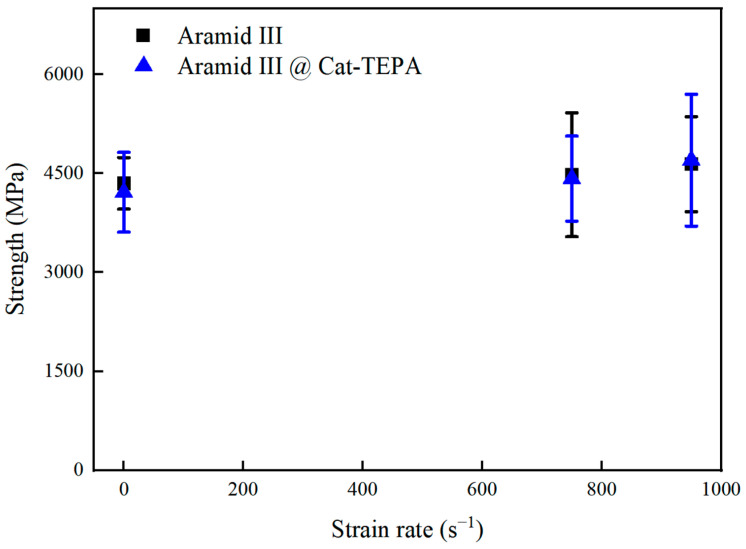
Tensile strengths of single Aramid III fibers before and after modification with Cat-TEPA at different strain rates.

Tensile tests were conducted on single Aramid III @ Cat-TEPA fibers at three strain rates: 0.001 s^−1^, 750 s^−1^, and 950 s^−1^, resulting in measured tensile strengths of 4214 MPa, 4422 MPa, and 4701 MPa, respectively. Figure 13 compares these results with experimental data from the literature, in which the tensile strength of the single Aramid III fibers at the strain rates of 0.001 s^−1^, 750 s^−1^, and 950 s^−1^ were 4350 MPa, 4480 MPa, and 4640 MPa, respectively [11]. The tensile strengths of the single Aramid III fibers before and after polyamine modification are illustrated in Figure 13. It is evident that the tensile mechanical properties of the single Aramid III fibers were not significantly affected by the Cat-TEPA modification. This can be attributed to the fact that the modification was conducted in water, at room temperature, under relatively mild conditions, thereby preserving the compositional structure of the modification. In comparison with the strong oxidizing agents and plasma treatment, the Cat-TEPA modification method offers significant advantages.

### 3.2. Interfacial Performance of Single Aramid III @ Cat-TEPA Fibers

**Figure 14:**
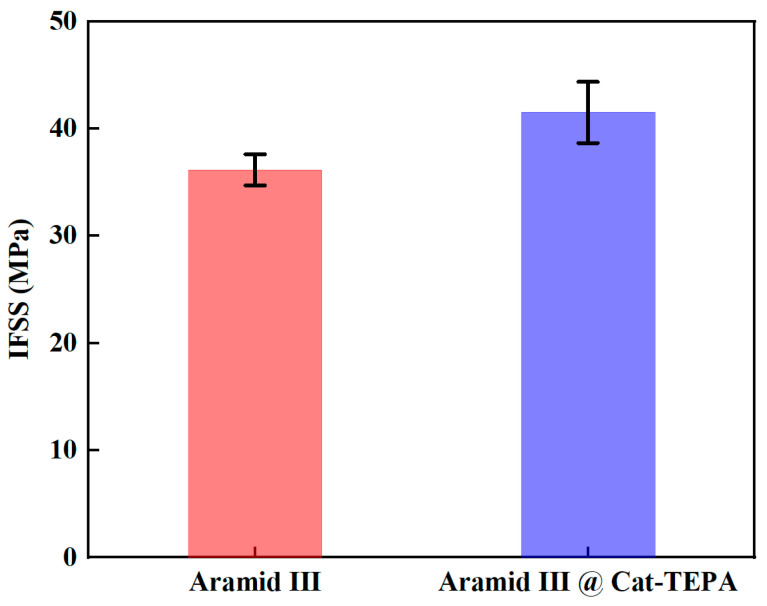
Dynamic IFSS of Aramid III and Aramid III @ Cat-TEPA.

The IFSS of Aramid III and Aramid III @ Cat-TEPA with waterborne polyurethanes were tested using the microdroplet method. The proposed impact speed of the sleeve-type bullet was 5 m/s. Before modification, the IFSS was measured at 36.16 MPa, whereas after modification, it increased to 41.51 MPa, indicating a 14.8% increase, as shown in Figure 14.

**Figure 15:**
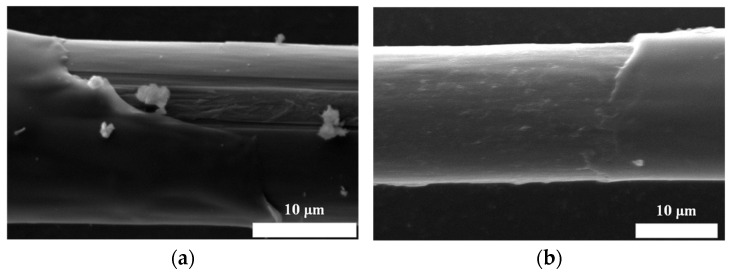
SEM morphology of microdroplet samples after the debonding test: (**a**) Aramid III; (**b**) Aramid III @ Cat-TEPA.

Figure 15 displays the SEM morphology of the Aramid III and Aramid III @ Cat-TEPA microdroplet samples after debonding. It is apparent that regular grid structures were observed on the debonding area of the single Aramid III @ Cat-TEPA fibers, which can effectively prevent the debonding of the fiber/resin interface, thereby contributing to an increase in the IFSS.

## 4. Conclusions

In this study, the polyamine modification of Aramid III fibers was conducted using the surface coating technique, and the dynamic mechanical properties of the single fibers and fiber/resin interface were tested utilizing the electronic tensile testing machine and mini SHTB, respectively. The following conclusions can be drawn:

(1) By incorporating a fixed energy-absorbing bar and an incident bar with a cutter device, improvements have been made to the mini SHTB, enabling the precise characterization of the dynamic mechanical properties of fiber/resin interfaces.
(2) The Cat-TEPA co-deposition method was utilized to prepare single Aramid III fibers, introducing amino functional groups on the fiber surface via the Cat-TEPA coating. This enhanced the properties of the fiber/resin interface.
(3) Quasi-static and dynamic tensile test results indicate that the Cat-TEPA modification method does not significantly affect the tensile strength of a single Aramid III fiber itself.
(4) A testing device based on the microdroplet technique for evaluating the dynamic mechanical properties of the fiber/resin interface was designed. The test results revealed that the dynamic shear strength of the fiber/resin interface of Aramid III @ Cat-TEPA was 41.51 MPa, representing a 14.8% increase compared to the strength before modification.

## Data Availability

Data are contained within the article.
